# Treatment of early stage Supraglottic squamous cell carcinoma: meta-analysis comparing primary surgery versus primary radiotherapy

**DOI:** 10.1186/s40463-018-0262-2

**Published:** 2018-03-05

**Authors:** Krupal B. Patel, Anthony C. Nichols, Kevin Fung, John Yoo, S. Danielle MacNeil

**Affiliations:** Department of Otolaryngology – Head & Neck Surgery, Schulich Medicine & Dentistry, London Health Sciences Centre, Western University, Victoria Hospital, London, ON Canada

**Keywords:** Early stage, Supraglottic squamous cell carcinoma, Supraglottic SCC, Outcomes, Meta-analysis

## Abstract

**Objectives:**

For early stage supraglottic squamous cell carcinoma (SCC), single modality treatment either in the form of primary organ preservation surgery alone or radiation alone is recommended. Thus, a definite treatment strategy for early stage supraglottic SCC remains undefined. The primary objective of this study was to conduct a systematic review and meta-analysis comparing the oncologic outcomes of surgery and radiotherapy in early stage (Stage I and II) T1 N0 and T2 N0 supraglottic SCC.

**Methods:**

Systematic methods were used to identify published and unpublished data. Two reviewers independently screened all titles, abstracts and articles for relevance using predefined criteria. Pooled odds ratios (ORs) and 95% confidence intervals (CIs) were calculated.

**Results:**

Five studies met the inclusion criteria for disease specific mortality with a total of 2864 pooled patients. 5-year disease specific mortality was lower in the surgery group (ORs 0.43, 95% CI 0.31–0.60). Four studies met the inclusion criteria for 5-year overall mortality with a total of 2790 pooled patients. Five-year overall mortality was lower in surgery group (ORs 0.40, 95% CI 0.29–0.55).

**Conclusions:**

This is the first study to examine the management of early stage supraglottic SCC using meta-analytic methodology. Our results suggest that primary surgery may result in decreased disease specific and overall mortality compared to primary radiotherapy.

## Background

Early stage supraglottic squamous cell carcinoma (SSCC) is defined as T1 (tumor limited to one subsite of supraglottis with normal vocal cord mobility) or T2 (tumor invading more than one adjacent subsite of supraglottis or glottis or region outside of supraglottis), with no regional nodal spread [1]. In a large review of nearly 160,000 cases of laryngeal SCC in the United States, the incidence of SSCC was noted to be 33% [2]. National Comprehensive Cancer Network (NCCN) guidelines for treatment of early stage SSCC suggest either organ preservation strategy – surgery with/without neck dissection or definitive radiation (RT) [3]. Early stage SSCC are small, however the 5-year survival of early stage SSCC is 64% [4, 5]. This is thought to be due to rich lymphatic supply of the area making it more likely to have occult regional and distant metastasis. Over the last 30 years, the oncologic outcomes for SSCC have not improved [2, 5, 6]. In fact, a review of National Cancer Database found that 5-year relative survival from SSCC decreased 52.2% (1985–1987) to 47.3% (1994–1996). The greatest decline in survival was in early stage SSCC patients with T1 N0 and T2 N0 disease.

Despite the poor survival of patients with early stage SSCC, there are a limited number of studies that have directly compared the survival outcomes of surgery versus radiation for early stage SSCC [7–16]. There are no prospective clinical trials, and the majority of the studies reported are small and retrospective. To date no meta-analysis comparing the survival outcomes for early stage SSCC comparing radiation and surgery has been reported. Our objectives were to, systematically review the literature to find all the relevant studies directly comparing surgery with radiation for early stage SSSC, synthesize the results and perform meta-analysis when possible of overall survival, disease specific survival and loco-regional control.

## Methods

A systematic review protocol was developed a priori to ensure the objectives and aims were outlined from the outset. This was approved by PROSPERO in November 2015 (CRD42015026590).

Randomized controlled trials, head-to-head comparative studies, observational studies, case series (greater than 3 patients) were assessed. Studies comparing surgery (open organ preservation (OPS), transoral endoscopic laser microsurgery (TLM) or transoral robotic surgery(TORS)) with/without neck dissection to definitive radiotherapy (RT) were included. Single arm studies that reported results of open surgery, transoral surgery alone or radiotherapy alone were not considered., due to the inherent selection biases and lack of ability to compare results between different modalities of treatment. The study population was limited to patients aged 18 and older and diagnosed with early stage supraglottic SCC (Tis, T1 N0, T2 N0).

Included studies were assessed for the following oncologic outcomes: 5-year overall mortality (OM); 5-year disease-specific mortality (DSM); 5-year local control (LC); 5-year laryngectomy free survival (LFS); and functional outcomes (quality of life, swallowing and voice quality).

Computerized bibliographic databases: Medline, EMBASE and Cochrane Central Register of Controlled Trials were searched to identify studies. English language records were included from January 1990 to May 2015. Search strategy was designed by two authors (K.B.P and S.D.M.) and an experienced librarian.

Two authors (K.B.P and S.D.M.) reviewed the titles, abstracts and full texts of the studies independently, with disagreements resolved by consensus. Interobserver agreement was analyzed with quadratic-weighted kappa. Titles were screened for the keywords: “squamous cell carcinoma” and “supraglottic”, or “supraglottis”, or “glottic”, or “glottis”, or “larynx”, or “laryngeal”. All abstracts of the studies that met the eligibility criteria were then screened. The full text of studies that met the criteria were then included. Newcastle-Ottawa quality assessment scale for cohort studies was used determine the quality of the studies [17]. The relevant data on outcome measures were extracted with the use of standardized data extraction forms. Not all studies contained data on all of the outcome measures.

Statistical Analysis was performed by Review Manager 5.3. Dichotomous outcomes were compared using odds ratios (OR) or weighted mean differences and 95% confidence intervals (CI). Heterogeneity across the studies was evaluated by the chi-square statistic and significance was set at *p* < 0.1. The I2 test was used to measure the extent of inconsistency among results. Fixed effects model was used given the assumption that included studies are only representative samples of all potentially available studies. The Z statistic was used to test for overall pooled effect and significance was set at *p* < 0.05.

## Results

**Fig. 1:**
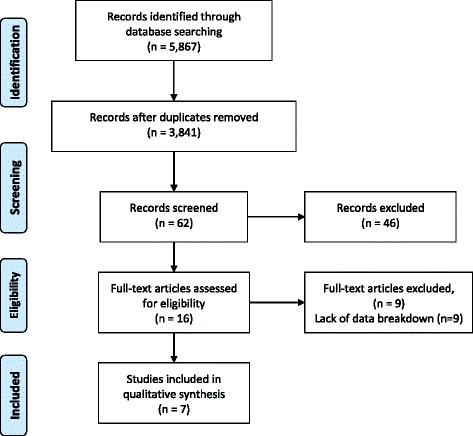
PRISMA Flowchart

The search strategy produced 5867 records. After removing 2026 duplicate records, the final number of unique records was 3841. After reviewing 3841 titles, 1098 studies were selected for reviewing the abstracts. Sixty-two abstracts were deemed appropriate for inclusion. After reviewing abstracts, 16 studies were deemed appropriate for inclusion and the full text was reviewed. Only 7 studies met the final inclusion criteria after reviewing the full text. Figure 1 illustrates the PRISMA (Preferred Reporting Items for Systematic Reviews and Meta-Analyses) flow chart to identify the appropriate studies. Kappa statistic for the agreement at the abstract screening stage was 0.57 (CI 0.46–0.67).

### Study characteristics and methodologic quality

No randomized controlled trials were found that compared the oncologic and functional outcomes of primary surgery versus RT. Of the seven studies included in the analysis, seven were retrospective and none were prospective in design. The total number of patients was 418 in the surgical arm, with patients undergoing organ preservation surgery with or without neck dissections. There were 2397 patients in the RT arm. Characteristics of the included studies are summarized in Table 1. Table 2 summarizes number of patients in each treatment group. Table 3 summarizes the quality of the included studies.

**Table 1:** Demographic characteristics of studies comparing survival outcomes between Surgery and Radiotherapy

Study	Median Age (years)		Gender (M/F)		Median Follow up (years)	
	RT	OPS	RT	OPS	RT	OPS
Arshad 2014 [7]	63	58	1476/802	119/48	NR	NR
Jones 2004 [10]	63^a^	61^a^	NR	NR	8.5	9.6
Orus 2000 [12]	63.5^a^	58.1^a^	NR	NR	1.5	
Santos 1998 [13]	56		NR	NR	NR	
Sessions 2005 [14]	N/A	N/A	NR	NR	> 5	
Spector 1995 [15]	61.3		41/12		NR	NR
Spriano 1997 [16]	62		141/16		5	

^a^numbers represent means Abbreviations: *RT* Radiotherapy, *OPS* Organ Preservation Surgery, *NR* Not Reported

**Table 2:** Staging characteristics of studies comparing survival outcomes between Surgery and Radiotherapy

**Study**	**RT**		**OPS +/− ND**	
	T1	T2	T1	T2
Arshad 2014 [7]	1043	1235	92	75
Jones 2004^a^ [10]	90		41	
Orus 2000^b^ [12]	27	63	11	14
Santos 1998^c^ [13]	3	6	3	6
Sessions 2005 [14]	5	5	90	102
Spector 1995^d^ [15]	4	1	1	2
Spriano 1997^a^ [16]	91		66	

*RT* Radiotherapy, *OPS* Organ Preservation Surgery, *ND* Neck Dissection

^a^T1 and T2 reported together, ^b^All OPS patients received ND, 7 patients received post-operative RT, ^c^Patients received post-operative RT, ^d^Only Aryepiglottic fold

**Table 3:** Quality of the studies reporting survival outcomes between Surgery and Radiotherapy (Newcastle-Ottawa Scale)^†^

Study	Selection				Comparability	Outcome			Score^*^
	Representativeness of exposed cohort	Selection of non exposed cohort	Ascertainment of exposure	Outcome not present at outset of study		Assessment	F/U Length	Adequacy of F/U	
Arshad 2014 [7]	*	*	*	*	**	*	*	*	9
Jones 2004 [10]	*	*	*	*	**	*	*	*	9
Orus 2000 [12]	*	*	*	*		*	*	*	7
Santos 1998 [13]	*	*	*	*	*	*	*		7
Sessions 2005 [14]	*	*	*	*		*	*	*	7
Spector 1995 [15]	*	*	*	*	**	*	*	*	9
Spriano 1997 [16]	*	*	*	*	*	*	*	*	8

^*^Maximum total score that a study can get is 9

^†^A study can receive a maximum of 1 asterisk for each numbered item within the selection and outcome categories. A maximum of 2 asterisks can be given for comparability

#### Oncologic outcomes

Of the seven studies included that were head-to-head studies, all seven contained data on oncologic outcome. Among them, data on overall survival were reported in four studies, data on disease specific survival were reported in five studies, data on local control were reported in one study.

Median age of the patients in the included studies was similar across the different studies. There were similar number of T1 and T2 patients within the RT and OPS groups for each study. There were higher number of patients in the RT group compared to the OPS +/− ND group.

### 5-year overall mortality (OM)

**Fig. 2:**
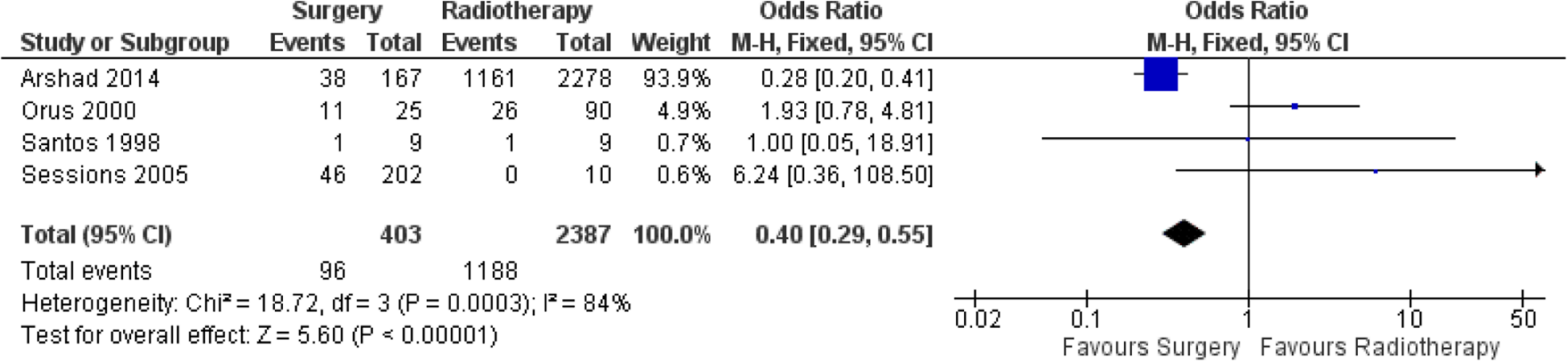
Forest Plot of comparison between organ preservation surgery and primary radiotherapy with respect to 5-year Overall Mortality

With respect to 5-year OM, in the head-to-head studies, there were 403 patients in the OPS with/without arm and 2387 patients in the RT arm in four studies. The results of pooled effect showed that the OR was 0.4 with 95% CI 0.29–0.55 favoring OPS with/without ND (Fig. 2).

### 5-year disease-specific mortality (DSM)

**Fig. 3:**
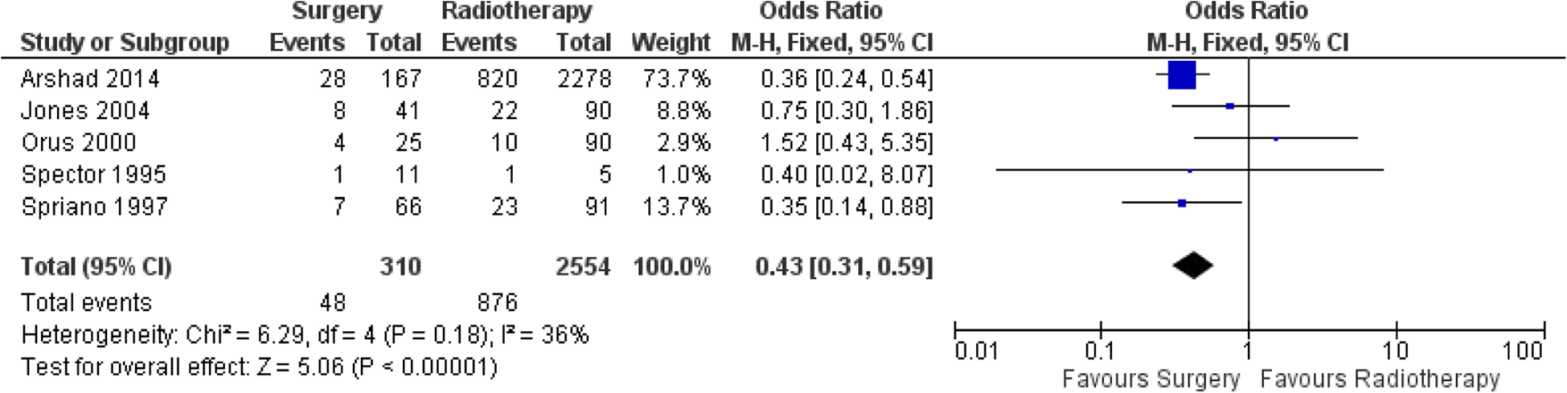
Forest Plot of comparison between organ preservation surgery and primary radiotherapy with respect to 5-year Disease-Specific Mortality

With respect to 5-year DSM, in the head-to-head studies, there were 310 patients in the OPS with/without ND arm and 2554 patients in the RT arm in five studies. The results of pooled effect showed that the OR was 0.43 with 95% CI 0.31–0.59 favoring OPS with/without ND (Fig. 3).

### 5-year local control (LC)

**Fig. 4:**
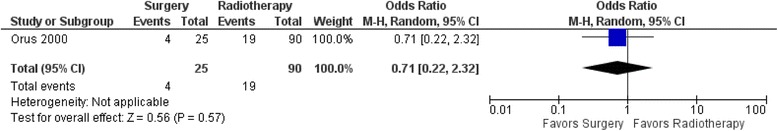
Forest Plot of comparison between organ preservation surgery and primary radiotherapy with respect to 5-year local recurrence

With respect to 5-year LC, in the head-to-head studies, there were 25 patients in the OPS arm and 90 patients in the RT arm in one study. The results of pooled effect showed that the OR was 0.71 with 95% CI 0.22–2.32 (Fig. 4).

### 5-year larynx preservation

No head-to-head studies were identified that compared the laryngeal preservation after surgery and radiotherapy.

### Functional outcomes

No head-to-head studies were identified that compared the functional outcomes after surgery and radiotherapy.

## Discussion

To our knowledge, this is the first meta-analysis comparing the survival outcomes of surgery versus radiotherapy for early-stage SSCC. Pooled analysis for 5-year OM favors OPS with/without ND over RT with OR of 0.4 (95% CI 0.29–0.55). These results however do need to be interpreted with caution as the heterogeneity was high amongst the studies with a significant *p*-value for the heterogeneity. Pooled analysis for 5-year DSM favors OPS with/without ND over RT with OR of 0.43 (95% CI 0.31–0.59). In this case the heterogeneity was low amongst the studies with a non-significant p-value for the heterogeneity, thus suggesting that these results are valid. Additionally, a jack-knife analysis was done to determine the validity of the results and to ensure that excessive contribution by one of the studies was not skewing the conclusions. The results of the jack-knife analysis produced similar results which were statistically significant and favoring OPS with/without ND. Only one study examined rate of local control which also showed better outcomes with OPS. Unfortunately, functional comparisons could not be made due to paucity of studies in the literature.

### Strengths

This review has several strengths. The review was designed, conducted and reported in accordance with published guidelines (PRISMA) and our protocol and search strategy was published a priori. To our knowledge, this is the first comprehensive review of all available literature comparing surgery versus radiation for patients with early stage SSCC. A comprehensive search strategy was undertaken and led to the review of 3841 unique citations of which seven studies met our inclusion criteria. This resulted in the analysis of 3086 patients with early stage SSCC.

### Limitations

There were no head-to-head studies that compared TLM or TORS to RT in oncologic or functional outcomes for early stage supraglottic cancer. As with all meta-analyses, the strength of the conclusions that can be drawn from this study depend on the quality of the primary studies. Although, we only included studies published from 1990 and onwards, some of the studies in our review included patients treated well before that time period. Due to poor quality of CT scanners before 1990, some patients may have had regional nodal disease (thus advanced stage disease) which was not apparent on a poor quality scan. Additionally, many of the contemporary modalities of treatment such intensity modulated radiotherapy (IMRT), Chemo-radiotherapy, TLM and TORS were not in clinical practice prior to 1990. All seven studies that met the inclusion criteria were retrospective, there were no randomized controlled trials. Retrospective studies have their inherent biases including selection biases, wherein patients with other health comorbidities would have been poor surgical candidates and would have likely received radiotherapy. Significant heterogeneity was noted between the studies. Not all studies included the type of radiation and the radiotherapy protocol used to treat these SSCC, the recruitment period for the patients was different which may have resulted in different radiotherapy protocols being used for the patients. In the surgical group, not all patients may have received the same extent of surgery including elective neck dissections. With regards to weight of the individual studies, Arshad et al. had the majority of the patients that were included in our analysis and thus their study was weighted proportionally larger skewing the results [7]. We only considered English language studies for our meta-analysis; this did limit the number of titles screened and studies included however, the effect of this would likely be small. OR were used for our statistical analysis as time to event (Hazard Ratios) could not be used given the lack of consistency in reporting the outcomes in the included studies.

### Surgery and radiotherapy for early stage SSCC

There are several advantages of RT. Although we did not find any head-to-head comparison studies assessing functional outcomes of RT in SSCC, it has been reported to have better functional outcomes in glottic cancers. Additionally, RT can be used in patients who are not candidates for OPS due to their underlying medical conditions. Risks of using RT in treating early stage SSCC is that this patient population does have a higher risk of developing a second primary malignancy in the aerodigestive tract [4, 5, 18]. If radiation is used as the primary treatment modality, most patients can only be salvaged with surgery and in the case of recurrent or new laryngeal cancer the treatment is almost always total laryngectomy.

Surgical approaches include open surgery or transoral surgical approaches, including laser (TLM) and robotic (TORS) and has several advantages over RT. As mentioned, patients with SSCC have a reasonable 5-year overall survival rates with a risk of developing second primary aerodigestive tract malignancy [4, 5, 18]. Surgery can thus be utilized as the first line and if there is failure, radiation can be used for salvage. Another advantage of surgery is the cost benefit of surgical intervention over radiotherapy. Cost analysis of open supraglottic laryngectomy, TLM and TORS by Dombree et al. in a Belgian model suggests that open supraglottic laryngectomy is almost equal to TLM in upfront surgical costs and TORS tends to be more expensive primarily due to purchase and maintenance costs [19]. This cost-analysis did not account for in-hospital costs such as length of admission, complications or readmission rates. Cost analysis comparing radiotherapy and TLM in a Canadian model for treatment of glottic cancers showed TLM to be a better cost saving modality [20].

One of the advantage of OPS with neck dissection is to identify patients with occult nodal metastasis in the neck. This is an important consideration given that up to 30% patients with SSCC may have occult nodal metastasis [21]. Thus, although these patients were early stage at the time of recruitment, the discovery of positive nodal metastasis after elective neck dissection results in the patients being upstaged and adjuvant radiotherapy is usually recommended. Some of the patients included in our study who underwent an elective neck dissection received adjuvant radiotherapy for positive nodal disease [12, 13]. This may in part be one of the reasons why patients in our study in the surgical arm had improved oncologic outcomes. Results from Arshad et al. corroborate this, as patients who underwent OPS with neck dissections fared better than those who only underwent RT or OPS without neck dissections [7].

Disadvantages of surgery include risk of general anesthetic, especially in patients with significant comorbidities, bleeding, infection, pharyngocutaneous fistula, dysphagia and tracheostomy. Additionally, one of the main criticisms of OPS are the associated poor functional outcomes [22]. However, TLM and TORS have gained popularity recently, due to several advantages of transoral surgery over open surgery and RT. TLM was first introduced by Strong and Jako for laryngeal surgery [23]. Since then several reports have been published investigating the role of TLM for supraglottic laryngectomy [23–36]. Long-term oncologic outcomes comparing TLM and open surgery suggest that oncologic outcomes are similar. Cabanillas et al. compared TLM versus open laryngeal preservation surgery in a total of fifty-two patients, who also underwent concurrent bilateral neck dissections, and found that 5-year DSS was 80% in the TLM group versus 72% in the open surgical group and 5-year local control rate was 70% in both groups [34]. Transoral laser surgery, when compared to open surgery, resulted in reduced incidence of permanent gastrostomies and tracheostomies [37]. Importantly, the survival outcomes were no different between the two groups.

TORS was first described by Weinstein and colleagues, and since then there have been several reports assessing its oncologic and functional outcomes, the majority of the studies report on all stages of supraglottic SCC [32, 38–49]. Although, long-term oncology outcomes have not been reported, initial results with mean follow up ranging from 6.8 to 28.1 months, indicate that locoregional control is the same as RT [38, 41, 44]. Additionally, long-term tracheostomy and gastric feeding tube rates range from 0 to 20% in patients treated with TORS [38, 41, 44].

Given the paucity of high level evidence guiding the optimal management of early stage supraglottic cancer and potential biases of retrospective studies, a head to head comparison between newer modalities such as TLM and/or TORS with RT is crucial in determining the therapeutic algorithm that can yield better oncologic and functional outcomes in early stage SSCC patient. Although studies comparing surgery and radiation have been challenging to accrue to, ongoing efforts comparing OPS to RT for oropharyngeal cancer are underway and actively accruing [50, 51]. This high level of evidence will ultimately be necessary to guide the guide treatment of these patients with early stage disease that have a surprisingly poor prognosis.

## Conclusions

To our knowledge, this is the first meta-analysis comparing RT and OPS for early stage SSCC. Patients who underwent OPS had better survival outcomes compared to primary radiation therapy. Five studies met the inclusion criteria for disease specific mortality with a total of 2864 pooled patients. 5-year disease specific mortality was lower in the surgery group (ORs 0.43, 95% CI 0.31–0.60). Four studies met the inclusion criteria for 5-year overall mortality with 5-year overall mortality being lower in surgery group (ORs 0.40, 95% CI 0.29–0.55). We were unable to compare the functional outcomes. Given the paucity of studies in the literature comparing open surgery, TLM, TORS and radiotherapy evaluating both oncologic and functions outcomes, future studies and research should include well designed randomized controlled trials.

## Abbreviations

CI: Confidence Intervals
DSM: Disease Specific Mortality
LC: Local Control
NCCN: National Comprehensive Cancer Network
ND: Neck Dissection
NR: Not Reported
OM: Overall Mortality
OPS: Organ Preservation Surgery
OR: Odds Ratios
PRISMA: Preferred Reporting Items for Systematic Reviews and Meta-Analyses
RT: Radiation Therapy
SSCC: Supraglottic squamous cell carcinoma
TLM: Transoral endoscopic laser microsurgery
TORS: Transoral robotic surgery
